# Associations between body composition and lifestyle factors with bone mineral density according to time since menopause in women from Southern Brazil: a cross-sectional study

**DOI:** 10.1186/s12902-015-0072-8

**Published:** 2015-11-21

**Authors:** Thaís R. Silva, Roberta Franz, Maria A. Maturana, Poli M. Spritzer

**Affiliations:** Gynecological Endocrinology Unit, Division of Endocrinology, Hospital de Clínicas de Porto Alegre (HCPA), Rua Ramiro Barcelos, 2350, 90035-003 Porto Alegre, RS Brazil; Laboratory of Molecular Endocrinology, Department of Physiology, Universidade Federal do Rio Grande do Sul (UFRGS), Rua Ramiro Barcelos, 2350, 90035-003 Porto Alegre, RS Brazil

**Keywords:** Menopause, Diet, Bone mass density, Lean mass, Osteoporosis, Lifestyle

## Abstract

**Background:**

The aim of this study was to investigate whether body composition, dietary pattern and habitual physical activity are associated with BMD according to time since menopause in women from Southern Brazil with no clinical evidence of disease.

**Methods:**

99 participants were enrolled and anthropometry, body composition and BMD by dual energy x-ray absorptiometry, rest metabolic rate by indirect calorimetry, dietary pattern by semi quantitative food frequency questionnaire and habitual physical activity by pedometer were performed.

**Results:**

Mean age was 55.2 ± 4.9 years and mean time since menopause was 6.8 ± 1.0 years. Weight, BMI, lean and fat mass and RMR were higher in women with less than 5 years since menopause with normal *versus* low bone mass. No differences were found in the studied variables between participants with normal or low bone mass and more than 5 years of menopause. Women with > 5 years since menopause had higher prevalence of osteoporosis, as well as lower BMD in all sites when compared to those with less time since menopause. Calories, carbohydrate, protein, fat and micronutrients intake were similar between groups. When the sample was adjusted for time since menopause, the odds ratio (OR) for low bone mass was 5.21 (95 % CI 1.57–17.25, *P* = 0.004) for BMI <25 kg/m^2^, for lean mass <37.5 Kg an OR of 4.4 (95 % CI 1.64–11.80, *P* = 0.004, for fat mass <26.0 Kg an OR of 3.39 (95 % CI 1.29–8.85, *P* = 0.010) and for the intake of vitamin A < 700 mcg/day an OR of 3.00 (95 % CI 1.13–7.94, *P* = 0.012). Low meat and eggs intake or low protein intake did not influence the odds ratio for low bone mass.

**Conclusion:**

In this cross-sectional study with postmenopausal women with no clinical evidence of disease, time since menopause, low lean and fat mass were associated with low bone mass. Calories and macronutrients intake as well as habitual physical activity did not interfere with BMD, but participants were mostly sedentary. Further studies are needed in order to determine whether the adequate intake of specific food groups and the type of physical activity could attenuate the time since menopause impact on BMD.

## Background

Bone mineral density (BMD) declines with increasing age, and the rate of decline is more pronounced after menopause [1]. Falling levels of 17-β-estradiol are thought to accelerate the decline in BMD, which remains the single best predictor of primary osteoporotic fracture [2]. This decline can also be attributed to a number of factors: age, genetics, nutrition, lifestyle factors, or the prolonged use of certain medication [3].

Body mass index (BMI) is known to be positively correlated with BMD, and low BMI (<19 kg/m^2^) significantly increases the risk of osteoporosis in postmenopausal women as compared to normal range BMI [4]. However, the contributions of lean and fat body mass to BMD, related to BMI *stratus*, are still not completely understood in different populations [5].

Lifestyle factors, such as physical activity (PA) and diet may exert influence on BMD in both pre- and postmenopausal women. PA plays a major role in minimizing bone loss as we age [6]. In addition, adequate dietary behavior seems to also influence on bone loss in postmenopausal women. In this sense, several studies had previously underline the importance of adequate calcium and Vitamin D levels in the prevention of osteoporosis and fractures in the peri- and post-menopause [7–10]. Besides that, studies have shown that diets with high content in vegetables, fruit, and whole grains may be associated with lower premenopausal bone loss in menopausal transition and lower risk of low-trauma fracture, particularly in older women [11, 12].

Therefore, the aim of this study was to investigate whether body composition, dietary pattern and habitual physical activity are associated with BMD according to time since menopause in women from Southern Brazil with no clinical evidence of disease.

## Methods

### Subjects

This cross-sectional study was carried out at the Gynecological Endocrinology Unit at Hospital de Clínicas de Porto Alegre, Brazil, from October 2010 to February 2012. Participants were recruited by advertisement in a local newspaper and radio station. Inclusion criteria were as follows: 1) menopause, defined as last menstrual period at least 1 year before the beginning of the study plus follicle stimulating hormone (FSH) levels > 35 IU/L; 2) age between 45 and 65 years; and 3) no use of hormone therapy in the past 3 months. Diabetic patients, patients with prior diagnosis of heart disease, and current smokers were excluded. These criteria were chosen because of the interest to study women with no clinically established systemic diseases. One hundred and nineteen postmenopausal women fulfilling all the inclusion criteria were consecutively enrolled. They were stratified by time since menopause (≤5 or > 5 years) and BMD (low or normal bone mass). The study protocol was approved by the local Research Ethics Committee from Hospital de Clinicas de Porto Alegre, and written informed consent was obtained from every participant.

### Design

All participants completed a questionnaire about their sociodemographic characteristics (e.g., age, education, household income, and marital status) and medical history (including current medications). The variable skin color was defined by auto-reference: participants were asked about their skin color and were stratified in white and no white. Anthropometric measurements were performed in duplicate and included body weight, height, and waist circumference [13]. BMI (kg/m^2^) was calculated. Resting metabolic rate (RMR) was obtained by indirect calorimetry (Fitmate®, Cosmed, Rome, Italy). Blood pressure was measured after resting for 10 min, in the sitting position. Two measurements were performed at a 10-min interval, using an automatic blood pressure monitor (Omron HEM 742, Rio de Janeiro, Brazil) with an appropriate cuff for the arm diameter. FSH, estradiol, total testosterone, sex-hormone binding globulin (SHBG), ultrasensitive C-reactive protein (us-CRP), total and high-density lipoprotein (HDL) cholesterol, triglycerides, fasting glucose and insulin were determined using the 12 h fasting blood sample. All samples were obtained between 8 AM and 10 AM, and were run immediately after collection. The methods of analysis did not change during the entire study.

### Assays

Total cholesterol, HDL cholesterol and triglycerides were determined by colorimetric-enzymatic methods (Bayer 1800 Advia System), with intra and interassay coefficients of variation (CV) < 3 %. Glucose was determined by the hexokinase method (Advia 1800) with intra-assay CV < 3.4 % and interassay CV < 2.1 %. FSH was measured by chemiluminescence immunoassay (CLIA) (Centaur XP), with sensitivity of 0.3 IU/L and intra and interassay CV of 2.9 and 2.7 % respectively. Total testosterone levels were also measured by CLIA (Centaur XP) with sensitivity of 10 ng/mL and intra and interassay CV of 3.3 and 7.5 % respectively. SHBG was measured by CLIA (Immulite 2000), with sensitivity of 0.02 nmol/L and intra- and interassay CV of 5.3 and 6.6 % respectively. Serum insulin levels were measured using CLIA (Centaur XP), with a sensitivity of 0.200 μIU/mL and intra- and interassay CV of 2.0 and 4.3 % respectively. FAI was estimated by dividing TT (in nanomoles per liter) by SHBG (in nanomoles per liter) × 100. Low-density lipoprotein (LDL) cholesterol was determined indirectly using the Friedewald formula LDL = total cholesterol – HDL – (triglycerides/5). Homeostasis model assessment (HOMA) was calculated by multiplying insulin (μIU/ml) by glucose (mmol/l) and dividing this product by 22.5.

### Bone mass and body composition assessments

BMD was assessed in lumbar spine (L1-L4), femoral neck and proximal total femur by dual-energy X-ray absorptiometry (DXA) (GE Lunar Prodigy, Radiation Corporation, Madison, WI, USA). BMD was expressed by g/cm^2^ and T-scores. Normal bone mass was defined as a T score above −1 standard deviations (SD) and low bone mass was defined as the presence of at least one site of osteopenia or osteoporosis, according to the World Health Organization (WHO) [14].

A whole body scan was also performed by DXA to assess body composition. Lean mass and fat mass were determined for the whole body, with a CV lower than 2 %.

### Dietary assessment

Usual dietary intake was assessed with a validated food frequency questionnaire consisting of 120 items [15]. Nutritional composition was calculated using the Brazilian Table of Food Composition [16] except for vitamin D, E, and A, which were assessed using the United States Department of Agriculture (USDA) National Standard Reference Database. Reference values for daily dietary intake were based on national [17] and international guidelines [18].

### Physical activity assessment

Assessment of habitual PA was performed with a digital pedometer (BP 148, Tech Line, São Paulo, Brazil). The device was configured individually according to weight (kg) and individual step length. The equipment was used for six consecutive days, providing a weekly average number of steps. Participants were stratified in active >6000 steps per day) or sedentary ≤ 6000 steps per day), according to previously reported [15, 19, 20]. Subjects were encouraged not to change their physical activity habits during the study.

### Sample size estimation and statistical analyses

Sample size was estimated based on a previous study [21], considering a power of 80 % and alpha of 5 %. One hundred women were required to detect a difference of 4.3 in BMI between women with normal and low bone mass.

Results are presented as mean ± standard deviation (SD), or median and inter-quartile range, depending on the Gaussian or non-Gaussian distribution of variables. Two-way ANOVA was used to assess the simultaneous effects of time since menopause and BMD. χ^2^ was calculated for comparisons of dichotomous variables. A logistic regression model was used to estimate the odds ratio of different variables forlow bone mass, which was considered as the dependent variable. All analyses were performed using the Statistical Package for the Social Sciences 19.0 (SPSS, Chicago, IL, USA). Data were considered to be significant at *p* < 0.05.

## Results

Of 119 volunteers, 13 were excluded (five with diabetes, one with hyperthyroidism, two with untreated hypothyroidism, two with breast cancer, one who was premenopausal and two with spinal disc prosthesis). An additional seven participants dropped out because they were unable to commit to the study (no time for blood collection, DXA and indirect calorimetry). Thus, 99 women were enrolled. Mean age was 55.2 ± 4.9 years and mean time since menopause was 6.8 ± 1.0 years. Participants had attended school for a mean of 8.5 ± 4.2 years, and 87 % were white. Forty participants were on antihypertensive drugs, two women were on statins, and one was taking aspirin.

**Fig. 1:**
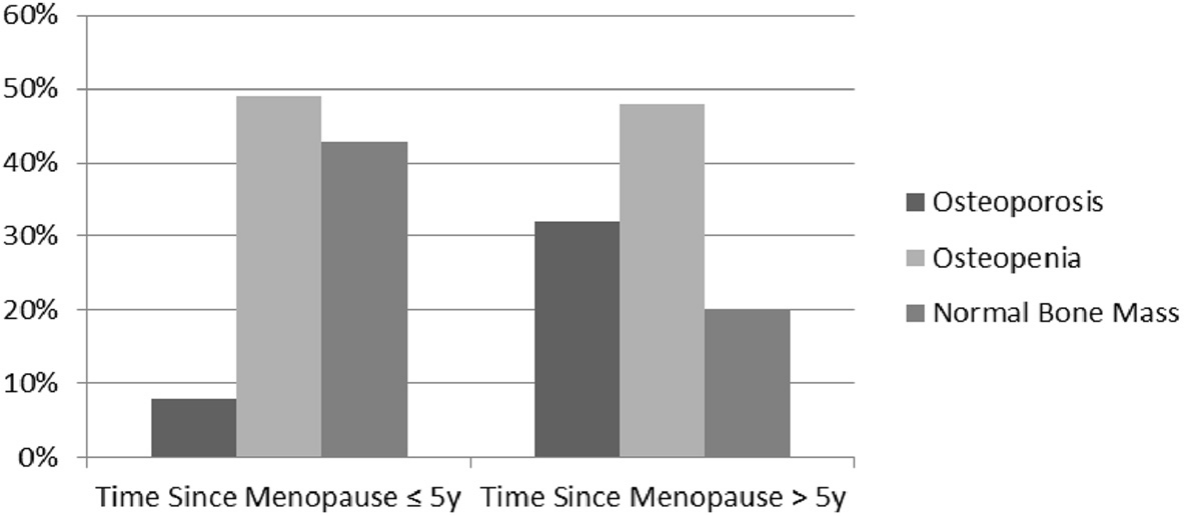
Prevalence of osteoporosis, osteopenia, and normal bone mass in women grouped according to time since menopause (≤5 and > 5 years)

Table 1 presents the demographic, hormonal and body composition characteristics of participants according to time since menopause (≤5 or > 5 years) and BMD (low or normal). The groups were similar regarding years at school, skin color, estradiol and free estradiol index. Number of steps per day, as an index of habitual physical activity was low and similar between groups and the prevalence of sedentary was around 50 and 70 % among groups. In turn, participants with ≤ 5 years since menopause presented higher weight, BMI, body fat %, lean mass, fat mass and RMR in the normal bone mass sub-group as compared to the group with low bone mass. Lumbar spine, femoral neck, and total femoral BMD were lower in both subgroups of low bone mass. Women with > 5 years since menopause and with low bone mass presented the lowest BMD in all sites in comparison with women with low bone mass and with ≤ 5 years. In addition, as shown in Fig. 1, women with > 5 years since menopause had also higher prevalence of osteoporosis.

**Table 1:** Characteristics of postmenopausal women according to time since menopause and bone mass *status*

	≤5 years since menopause		>5 years since menopause				
	Normal bone mass	Low bone mass	Normal bone mass	Low bone mass	*P* value		
	(*n* = 21)	(*n* = 28)	(*n* = 10)	(*n* = 40)	Time since menopause	BMD	T x B^1^
Age (years)	52.2 ± 4.2^a^	52.1 ± 3.3^a^	55.9 ± 4.4^b^	58.9 ± 3.8^c^	**<0.001**	0.096	0.076
Years at school (years)	9.5 ± 3.4	8.3 ± 4.5	6.2 ± 3.3	8.5 ± 4.7	0.120	0.535	0.079
White, *n* (%)^d^	19 (90)	24 (86)	8 (80)	35 (87)		0.688	
Weight (kg)	77.9 ± 15.2^b^	62.7 ± 8.6^a^	69.5 ± 9.4^b^	65.5 ± 11.0^ba^	0.282	**<0.001**	**0.033**
BMI (kg/m^2^)	30.6 ± 5.8^b^	25.6 ± 2.9^a^	27.4 ± 3.5^b^	26.3 ± 4.3^ba^	0.189	**0.002**	0.050
Waist circumference (cm)	95.4 ± 14.8^b^	82.5 ± 7.9^a^	87.6 ± 8.9^b^	84.4 ± 10.7^ba^	0.239	**0.002**	0.054
Body fat %	44.2 ± 5.6^b^	39.5 ± 6.2^a^	41.3 ± 4.8^b^	38.9 ± 6.2^ba^	0.262	**0.025**	0.453
Lean mass (kg)	41.3 ± 5.9^b^	35.6 ± 4.2^a^	38.7 ± 5.3^b^	37.7 ± 3.8^ba^	0.797	**0.002**	**0.027**
Fat mass (kg)	33.7 ± 10.3^b^	25.2 ± 9.6^a^	27.5 ± 6.2^b^	25.0 ± 8.7^ba^	0.131	**0.010**	0.159
RMR (Kcal/day)	1358.4 ± 285.8^b^	1191.8 ± 134.8^a^	1302.0 ± 189.8^b^	1242.5 ± 165.3^ba^	0.949	**0.012**	0.227
Mean steps/day	3681.6 ± 208.8	3718.9 ± 262.3	3659.1 ± 225.7	3731.0 ± 243.8	0.925	0.323	0.754
Sedentary, *n* (%)^d^	14 (67)	16 (57)	7 (70)	21 (52)		0.564	
Lumbar spine BMD (g/cm^2^)	1.20 ± 0.08^c^	1.00 ± 0.08^b^	1.19 ± 0.09^c^	0.94 ± 0.13^a^	0.139	**<0.001**	0.352
Femoral neck BMD (g/cm^2^)	1.01 ± 0.08^c^	0.88 ± 0.08^b^	0.98 ± 0.07^c^	0.80 ± 0.09^a^	**0.027**	**<0.001**	0.344
Total femoral BMD (g/cm^2^)	1.07 ± 0.09^c^	0.92 ± 0.09^b^	1.02 ± 0.08^c^	0.86 ± 0.10^a^	**0.015**	**<0.001**	0.744
Estradiol (pg/mL)	27.6 ± 15.7	22.2 ± 12.6	20.9 ± 10.4	20.1 ± 11.9	0.178	0.191	0.583
Free estradiol index (pg/mL)	0.4 ± 0.2	0.3 ± 0.2	0.3 ± 0.1	0.3 ± 0.2	0.178	0.191	0.583

Values are expressed as mean ± SD, median and 25–75 inter-quartile range or absolute and percentage number

*BMI* body mass index, *RMR* resting metabolic rate, *BMD* bone mineral density. ^1^T × B = Time since menopause × Bone Mineral Density interaction effect. ^a-c^Means in a row without a common superscript letter differ (*P* < 0.05), as analyzed by two-way ANOVA and Bonferroni test. ^d^Qui-square Test. *p* values in boldface reflect statistical significance

Calories, carbohydrate, protein, fat and micronutrients intake were similar between groups (data not presented). Vitamin A intake was greater in the groups with normal bone mass compared to groups with low bone mass, with a borderline significance (1239.7 ± 778.6 *vs* 926.8 ± 819.0 for groups with ≤ 5 years since menopause and 1363.4 ± 1199.4 *vs* 895.1 ± 871.0 for those with >5 years of menopause; *P* = 0.051).

Table 2 shows lumbar spine, femoral neck and total femoral BMD in postmenopausal women according to different factors, stratified by tertiles. Age, time since menopause, fat mass, and RMR were associated with BMD in all sites. BMI was associated with BMD on femoral neck and total femoral but not in the lumbar spine. None dietary or hormonal variables were associated with BMD (data not presented).

**Table 2:** Lumbar spine, femoral neck and total femoral bone mineral density in postmenopausal women according to factors

Factor	Tertile	*N*	Lumbar spine BMD (g/cm^2^)	*P*	Femoral neck BMD (g/cm^2^)	*P*	Total femoral BMD (g/cm^2^)	*P*
Age (years)	≤53	37	1.11 ± 0.13^a^	**0.002**	0.94 ± 0.11^a^	**<0.001**	1.00 ± 0.11^a^	**<0.001**
	53–58	31	1.00 ± 0.15^b^		0.88 ± 0.11^a^		0.93 ± 0.11^b^	
	≥58	31	0.98 ± 0.14^b^		0.81 ± 0.09^b^		0.88 ± 0.11^b^	
Time since menopause (years)	≤3	34	1.08 ± 0.13^a^	**0.002**	0.91 ± 0.11^a^	**0.008**	0.98 ± 0.12^a^	**0.002**
	3–8	32	1.07 ± 0.15^a^		0.90 ± 0.11^a^		0.96 ± 0.11^a^	
	≥8	33	0.96 ± 0.15^b^		0.83 ± 0.11^b^		0.88 ± 0.12^b^	
BMI (kg/m^2^)	≤25	33	1.00 ± 0.13	0.086	0.82 ± 0.10^a^	**0.001**	0.87 ± 0.10^a^	
	25–28.2	34	1.04 ± 0.14		0.91 ± 0.10^b^		0.97 ± 0.10^b^	**<0.001**
	≥28.2	32	1.08 ± 0.17		0.91 ± 0.12^b^		0.98 ± 0.13^b^	
Fat Mass (kg)	≤23.4	33	1.01 ± 0.13^a^	**0.005**	0.84 ± 0.12^a^	**0.016**	0.89 ± 0.1 ^a^	**0.003**
	23.4–29.8	34	1.01 ± 0.17^a^		0.89 ± 0.12^ab^		0.94 ± 0.13^ab^	
	≥29.8	32	1.11 ± 0.13^b^		0.92 ± 0.10^b^		0.99 ± 0.110^b^	
Lean Mass (kg)	≤36.0	34	1.02 ± 0.15	0.108	0.86 ± 0.10		0.92 ± 0.10	
	36.0–39.2	33	1.02 ± 0.14		0.87 ± 0.12	0.086	0.93 ± 0.13	0.130
	≥39.2	32	1.08 ± 0.15		0.92 ± 0.12		0.98 ± 0.12	
Estradiol (pg/mL)	≤15	34	1.00 ± 0.14	0.252	0.85 ± 0.12		0.91 ± 0.13	0.102
	15–26	33	1.04 ± 0.16		0.88 ± 0.12	0.069	0.94 ± 0.13	
	≥26	32	1.07 ± 0.15		0.91 ± 0.10		0.97 ± 0.10	
Free estradiol (pg/mL)	≤0.20	33	0.99 ± 0.14	0.207	0.84 ± 0.12^a^	0.053	0.91 ± 0.13	0.093
	0.2–0.35	32	1.04 ± 0.15		0.89 ± 0.12^ab^		0.94 ± 0.13	
	≥0.35	34	1.07 ± 0.15		0.91 ± 0.10^b^		0.97 ± 0.10	
RMR (Kcal)	≤1175	33	1.01 ± 0.15^a^	**0.005**	0.85 ± 0.11^a^	**0.046**	0.90 ± 0.10	**0.028**
	1175–1324	35	1.00 ± 0.14^a^		0.89 ± 0.11^ab^		0.95 ± 0.13	
	≥1325	31	1.01 ± 0.13					

^a-b^Means in a column without a common superscript letter differ (*P* < 0.05), as analyzed by one-way ANOVA and the Bonferroni test. *p* values in boldface reflect statistical significance

Compared with women who underwent menopause ≤5 years, those who underwent menopause >5 years ago had a 3-fold increase in odds ratio for low bone mass (95 % CI 1.27–7.34, *P* = 0.016). However, being older than 55 years (mean age of participants in this sample) did not increase the odds ratio for low bone mass. Therefore, when adjusted for time since menopause and previous hormone therapy the odds ratio (OR) for low bone mass was 5.21 (95 % CI 1.57–17.25, *P* = 0.004), for BMI <25 kg/m^2^, for lean mass <37.5 Kg an OR of 4.4 (95 % CI 1.64–11.80, *P* = 0.004, for fat mass <26.0 Kg an OR of 3.39 (95 % CI 1.29–8.85, *P* = 0.010) and for the intake of vitamin A < 700 mcg/day an OR of 3.00 (95 % CI 1.13–7.94, *P* = 0.012). Low meat and eggs intake or low protein intake (defined as the median consumption of participants in this sample) did not influence the odds ratio for low bone mass (Table 3).

**Table 3:** Odds ratio for low bone mass

Variables	OR	95 % CI	*P* ^*^
BMI (<25 kg/m^2^)^a^	**5.21**	**1.57 – 17.25**	**0.004**
Lean Mass (<37.5 kg)^b^	**4.40**	**1.64 – 11.80**	**0.004**
Fat Mass (<26.0 kg)^b^	**3.39**	**1.29 – 8.85**	**0.010**
Vitamin A (<700 mcg/day)^c^	**3.00**	**1.13 – 7.94**	**0.012**
Meat and eggs (<96 g/day)^b^	2.30	0.90 – 5.86	0.081

*BMI* body mass index. ^*^Logistic regression adjusted for time since menopause and previous hormonal therapy. ^a^Defined as the first tertile of the studied sample; ^b^defined as the median of participants in this sample; ^c^Dietary Reference Intake (2002) values in boldface reflect statistical significance

## Discussion

In the present study, weight, BMI, lean and fat mass and RMR were higher in postmenopausal women with less than 5 years since menopause with normal *versus* low bone mass. Interestingly, these variables did not differ significantly between women with normal and low bone mass and more than 5 years since menopause. This observation seems to support previous evidence showing that BMD is better correlated with percentage of body fat in pre- and perimenopausal than in postmenopausal women [22]. In addition, while it is known that total BMD is associated with higher BMI and fat mass, the profile of trabecular and cortical volumetric BMD (vBMD) varies according to BMI. Indeed, obese adults present higher trabecular vBMD but lower cortical vBMD [23]. Therefore, we hypothesize that the association of adiposity and BMD was more evident in recent postmenopausal women because they had more trabecular bone than those in later postmenopausal life (in whom trabecular bone is lost due to the high bone turnover occurring throughout the postmenopausal years). Further studies assessing volumetric bone density and microarchitecture by high-resolution peripheral quantitative computed tomography in postmenopausal populations are needed to confirm this hypothesis.

When the entire sample was analyzed, age, time since menopause, fat mass and RMR were associated with BMD in all three sites.

In fact, menopause is associated with a few years of rapid bone loss attributed to lower circulating levels of 17β-estradiol, related primarily to the decline in estrogen-mediated inhibition of bone resorption without a fully compensatory increase in bone formation [2]. For an interval of few years around the menopause, women lose 2 % of bone annually. Afterward, bone loss slows to about 1 to 1.5 % per year [24, 25]. Recker et al., [25] found that menopausal bone loss is a composite of loss caused by estrogen deprivation and age *per se* for the hip and total body, but is caused by estrogen deprivation alone for the spine. In the Study of Women’s Health Across the Nation (SWAN), women who transitioned through menopause experienced a significantly higher rate of bone loss than women who remained premenopausal, independent of age [26]. In turn, a recent study suggests that time since menopause may have a stronger predictive value for low BMD in the lumbar and hip areas than did serum FSH or estradiol levels [27]. Data from the present study reinforces that idea, showing that women with more than 5 years since menopause had higher prevalence of osteoporosis, as well as lower BMD in all sites when compared to those with less time since menopause. In addition, women who underwent menopause more than 5 years ago had a 3-fold increased odds ratio for low bone mass.

Regarding the association between BMD and BMI we found in our study, the odds ratio for low bone mass was five times for a BMI lower than 25 kg/m^2^, being this cut-off the lowest tertile of our sample. In this sense, low weight or low BMI is a well-documented risk factor for future fracture [1]. Zhu and coworkers have recently reported in a Western Australian population that the associations of BMI with BMD measures were attenuated in those with high BMI [5], suggesting that low body weight should be considered as a risk factor for osteoporosis and related fracture, rather than obesity being a protective factor.

In the present study fat mass was associated with BMD in all sites and with reduced odds ratio for low bone mass. However, the influence of fat mass on BMD is a debatable issue and seems to be related to menopausal status [28]. In postmenopausal women, adipose tissue is the major sources of estrogen from aromatization [29]. Therefore, it has been suggested that subcutaneous adipose tissue have higher aromatase activity in comparison to visceral adipose tissue, and could exert a more beneficial effect than visceral fat in bone health after menopause. In this sense, body composition analysis by DXA does not allow to discriminate subcutaneous and visceral fat, which is a limitation of the present study. Further studies using other methodologies are needed in order to clarify this issue.

While in our study BMD measures in all three sites did not differ according to lean mass tertiles, lean mass was lower in participants with low bone mass *versus* normal bone mass and less than 5 years since menopause. Lima and coworkers showed that in older women, lean mass was significantly correlated with BMD independently of height and fat mass [28]. Some other cross sectional studies with postmenopausal women suggests that lean mass is not an independent correlate of BMD [30, 31]. In turn, in pre- and peri-menopausal women lean mass has been reported to be a main predictor of BMD [32, 33]. The stronger association between lean mass and BMD may be attributed to differences in determinants of lean mass, such as exercise, lifestyle factors, serum estrogen concentrations or a combination of these factors [32].

Concerning RMR, studies in different populations have also reported a strong relationship between BMD and RMR [34, 35]. We found that participants with low bone mass have lower RMR, in line with a previous research that have also shown that a lower lean mass in postmenopausal women is associated with a lower RMR [36]. Taken together these data suggest that interventions aiming to increase lean mass, which increases RMR, could represent a simple and useful strategy to prevent osteoporosis in women, especially in recent postmenopausal women, such as physical activity (PA) practice. In fact, intervention studies have reported positive effects or associations between PA, BMD and markers of bone metabolism in pre- and postmenopausal women [37, 38]. However, walking may not be enough as a stimulus to increase lean mass in postmenopausal women [39], and these women should be encouraged to participate in regular programs of moderate physical activity [40]. Indeed, in the present study, participants were mostly sedentary, as objectively estimated by a pedometer, and this could have influenced on the association between lean mass and BMD that was independent of habitual PA.

In the specific context of osteoporosis prevention and management, a discussion of nutrition appropriately focuses on vitamin D, and protein in addition to calcium. According to our data the mean calcium intake was 799 mg/day meaning 69 % of dietary reference intakes for American women (1200 mg/day). However, in a longitudinal and prospective cohort study, based on the Swedish MammographyCohort [39] only calcium intake lower than 700 mg/day increased risk of fractures and of osteoporosis, high levels of intake did not further decrease the rate of fracture [41]. In the same cohort although not reflected in the fracture rate, women with high vitamin D intake (>5.4 μg/day) tended to have a slightly higher BMD, but in the present study vitamin D (mean of intake 4 μg/day) did not influence the BMD. In a recently published reanalysis of randomized vitamin D supplementation trials, it was concluded that an intake of 20 μg/day is needed to prevent nonvertebral fractures in women and men aged 65 years or older [6]. Regarding calories intake, a previous study showed higher cortical BMD in elderly women (mean age of 75 years) eating a diet exceeding the RDA for macronutrients (44 kcal/kg of ideal body weight) [42]. Interestingly, we did not find any association between calories intake and BMD, probably because our participants were in early postmenopausal. Dietary protein is positively linked to the maintenance of bone and muscle health and some experts suggest that the current recommended protein intake (≈70 g/day) may be inadequate for optimum skeletal and muscle health [43]. Our postmenopausal women had an average protein intake of 77 g/day, which may not be sufficient for interfering with the risk for low bone mass, as shown by the neutral OR related to meat and eggs food intake.

In turn, when micronutrients intake was analyzed, only vitamin A appears to be less consumed among women with lower bone mass. Considering all sample, vitamin A intake lower than 700mcg a day, that is the recommended amount for dietary reference intakes [18], was related to a higher OR for low bone mass. Indeed, vitamin A, retinol, beta‐carotene, and its metabolites are involved in bone metabolism and Wattanapenpaiboon et al*.* have shown a positive correlation of lumbar BMD with β -carotene levels in postmenopausal women [44]. A recent meta-analysis, also reported an U‐shaped relationship between serum retinol levels and hip fracture risk [45].

One limitation of the present study is the cross-sectional design that does not allow conclusions regarding the direction of cause and effect. Other limitation is the relatively small sample size of 99 participants and a moderate enrollment rate (16 % excluded participants), which could affect the external validity. However, the results observed in our sample of Southern Brazilian postmenopausal women are consistent and in line to those reported in other populations. Another limitation is that although pedometers are increasingly used to estimate habitual physical activity, they are not sensitive to the intensity or the type of the activity performed, and therefore may not accurately depict the loading forces of the activities performed, which are important for bone maintenance and/or development [38].

## Conclusions

In postmenopausal women from Southern Brazil, with no clinical evidence of disease, time since menopause, low lean and fat mass were associated with low bone mass. Calories and macronutrients intake as well as habitual physical activity did not interfere with BMD, but participants were mostly sedentary. Further studies are needed in order to determine whether the adequate intake of specific food groups and the type of physical activity could attenuate the aging and time since menopause impact on BMD.

## Abbreviations

BMD: bone mineral density
BMI: bone mass index
DHEAS: dehydroepiandrosterone sulfate
DXA: dual-energy X-ray absorptiometry
FAI: free androgen index
FSH: follicle-stimulating hormone
PA: physical activity
RMR: resting metabolic rate
SHBG: sex-hormone binding globulin
us-CRP: ultrasensitive C-reactive protein

## References

[1] Cummings SR, Nevitt MC, Browner WS, Stone K, Fox KM, Ensrud KE (1995). Risk factors for hip fracture in white women. Study of Osteoporotic Fractures Research Group. N Engl J Med.

[2] Riggs BL, Khosla S, Melton LJ (1998). A unitary model for involutional osteoporosis: estrogen deficiency causes both type I and type II osteoporosis in postmenopausal women and contributes to bone loss in aging men. J Bone Miner Res.

[3] Guthrie JR, Ebeling PR, Hopper JL, Barrett-Connor E, Dennerstein L, Dudley EC (1998). A prospective study of bone loss in menopausal Australian-born women. Osteoporos Int.

[4] The North American Menopause Society (2010). Management of osteoporosis in postmenopausal women. Menopause.

[5] Zhu K, Hunter M, James A, Lim EM, Walsh JP (2015). Associations between body mass index, lean and fat body mass and bone mineral density in middle-aged Australians: The Busselton Healthy Ageing Study. Bone.

[6] Kohrt WM, Bloomfield SA, Little KD, Nelson ME, Yingling VR (2004). American College of Sports Medicine Position Stand: physical activity and bone health. Med Sci Sports Exerc.

[7] DIPART (Vitamin D Individual Patient Analysis of Randomized Trials) Group (2010). Patient level pooled analysis of 68500 patientes from seven major vitamin D fracture trials in US and Europe. BMJ.

[8] Avenell A, Gillespie WJ, Gillespie LD, O’Connell D (2009). Vitamin D and vitamin D analogues for preventing fractures associated with involutional and post-menopausal osteoporosis. Cochrane Dataset Syst Rev.

[9] Tang BM, Eslick GD, Nowson C, Smith C, Bensoussan A (2007). Use of calcium or calcium in combination with vitamin D supplementation to prevent fractures and bone loss in people aged 50 years and older: a meta-analysis. Lancet.

[10] Bischoff-Ferrari HA, Dawson-Hughes B, Baron JA, Burckhardt P, Li R, Spiegelman D (2007). Calcium intake and hip fracture risk in men and women: a meta-analysis of prospective cohort studies and randomized controlled trials. Am J Clin Nutr.

[11] Macdonald HM, New SA, Golden MH, Campbell MK, Reid DM (2004). Nutritional associations with bone loss during the menopausal transition: evidence of a beneficial effect of calcium, alcohol, and fruit and vegetable nutrients and of a detrimental effect of fatty acids. Am J Clin Nutr.

[12] Langsetmo L, Hanley DA, Prior JC, Barr SI, Anastassiades T, Towheed T (2011). Dietary patterns and incident low-trauma fractures in postmenopausal women and men aged ≥ 50 y: a population-based cohort study. Am J Clin Nutr.

[13] Donato GB, Fuchs SC, Oppermann K, Bastos C, Spritzer PM (2006). Association between menopause status and central adiposity measured at different cutoffs of waist circumference and waist-to-hip ratio. Menopause.

[14] Kanis JA, Melton LJ, Christiansen C, Johnston CC, Khaltaev N (1994). The diagnosis of osteoporosis. J Bone Miner Res.

[15] Silva TR, Maturana M, Spritzer PM (2013). Healthier dietary pattern and lower risk of metabolic syndrome in physically active postmenopausal women. J Am Coll Nutr.

[16] Center for Studies and Research in Food: Brazilian table of food composition. Campinas: NEPA-UNICAMP; 2011. http://www.unicamp.br/nepa/taco/contar/taco_4_edicao_ampliada_e_revisada

[17] Fernandes CE, Pinho-Neto JSL, Gebara OCE, Santos Filho RD, Pinto Neto AM (2008). I Diretriz Brasileira sobre Prevenção de Doenças Cardiovasculares em Mulheres Climatéricas e a Influência da Terapia de Reposição Hormonal (TRH) da Sociedade Brasileira de Cardiologia (SBC) e da Associação Brasileira do Climatério (SOBRAC). Arq Bras Cardiol.

[18] Trumbo P, Schlicker S, Yates AA, Poos M (2002). Dietary reference intakes for energy, carbohydrate, fiber, fat, fatty acids, cholesterol, protein and amino acids. J Am Diet Assoc.

[19] Graff SK, Alves BC, Toscani MK, Spritzer PM (2012). Benefits of pedometer-measured habitual physical activity in healthy women. Appl Physiol Nutr Metab.

[20] Colpani V, Oppermann K, Spritzer PM (2013). Association between habitual physical activity and lower cardiovascular risk in premenopausal, perimenopausal, and postmenopausal women: a population-based study. Menopause.

[21] Sheng Z, Xu K, Ou Y, Dai R, Luo X, Liu S (2011). Relationship of body composition with prevalence of osteoporosis in central south Chinese postmenopausal women. Clin Endocrinol.

[22] Lindsay R, Cosman F, Herrington BS, Himmelstein S (1992). Bone mass and body composition in normal women. J Bone Miner Res.

[23] Sukumar D, Schlussel Y, Riedt CS, Gordon C, Stahl T, Shapses SA (2011). Obesity alters cortical and trabecular bone density and geometry in women. Osteoporos Int.

[24] Pouilles JM, Tremollieres F, Ribot C (1996). Vertebral bone loss in perimenopause: results of a 7-year longitudinal study. Presse Med.

[25] Recker RR, Lappe J, Davies K, Heaney R (2000). Characterization of perimenopausal bone loss: a prospective study. J Bone Miner Res.

[26] Finkelstein JS, Brockwell SE, Mehta V, Greendale GA, Sowers MR, Ettinger B (2008). Bone mineral density changes during the menopause transition in a multiethnic cohort of women. J Clin Endocrinol Metab.

[27] Yoldemir T, Erenus M, Durmusoglu F (2012). The impact of serum FSH and estradiol on postmenopausal osteoporosis related to time since menopause. Gynecol Endocrinol.

[28] Lima RM, Bezerra LM, Rabelo HT, Silva MA, Silva AJ, Bottaro M (2009). Fat-free mass, strength, and sarcopenia are related to bone mineral density in older women. J Clin Densitom.

[29] Simpson ER (2003). Sources of estrogen and their importance. J Steroid Biochem Mol Biol.

[30] Reid IR, Ames R, Evans MC, Sharpe S, Gamble G, France JT (1992). Determinants of total body and regional bone mineral density in normal postmenopausal women-a key role for fat mass. J Clin Endocrinol Metab.

[31] Nur H, Toraman NF, Arica Z, Sarier N, Samur A (2013). The relationship between body composition and bone mineral density in postmenopausal Turkish women. Rheumatol Int.

[32] Salamone LM, Glynn N, Black D, Epstein RS, Palermo L, Meilahn E (1995). Body composition and bone mineral density in premenopausal and early perimenopausal women. J Bone Miner Res.

[33] Li S, Wagner R, Holm K, Lehotsky J, Zinaman MJ (2004). Relationship between soft tissue body composition and bone mass in perimenopausal women. Maturitas.

[34] Kaufman BA, Warren MP, Dominguez JE, Wang J, Heymsfield SB, Pierson RN (2002). Bone density and amenorrhea in ballet dancers are related to a decreased resting metabolic rate and lower leptin levels. J Clin Endocrinol Metab.

[35] Doyle-Lucas AF, Akers JD, Davy BM (2010). Energetic efficiency, menstrual irregularity, and bone mineral density in elite professional female ballet dancers. J Dance Med Sci.

[36] Hodson L, Harnden K, Banerjee R, Real B, Marinou K, Karpe K (2014). Lower resting and total energy expenditure in postmenopausal compared with premenopausal women matched for abdominal obesity. J Nutr Sci.

[37] Heinonen A, Oja P, Sievanen H, Pasanen M, Vuori I (1998). Effect of two training regimens on bone mineral density in healthy perimenopausal women: a randomized controlled trial. J Bone Miner Res.

[38] Yamazaki S, Ichimura S, Iwamoto J, Takeda T, Toyama Y (2004). Effect of walking exercise on bone metabolism in postmenopausal women with osteopenia/osteoporosis. J Bone Miner Metab.

[39] Hinriksdottir G, Arngrimsson SA, Misic MM, Evans EM (2013). Lean soft tissue contributes more to bone health than fat mass independent of physical activity in women across the lifespan. Maturitas.

[40] Wee J, Sng BY, Shen L, Lim CT, Singh G, De Das S (2013). The relationship between body mass index and physical activity levels in relation to bone mineral density in premenopausal and postmenopausal women. Arch Osteoporos.

[41] Warensjo E, Byberg L, Melhus H, Gedeborg R, Mallmin H, Wolk A (2011). Dietary calcium intake and risk of fracture and osteoporosis: prospective longitudinal cohort study. BMJ.

[42] Pedone C, Napoli N, Pozzilli P, Rossi FF, Lauretani F, Bandinelli S (2011). Dietary pattern and bone density changes in elderly women: a longitudinal study. J Am Coll Nutr.

[43] Heaney RP, Layman DK (2008). Amount and type of protein influences bone health. Am J Clin Nutr.

[44] Wattanapenpaiboon N, Lukito W, Wahlqvist ML, Strauss BJ (2003). Dietary carotenoid intake as a predictor of bone mineral density. Asia Pac J Clin Nutr.

[45] Wu AM, Huang CQ, Lin ZK, Tian NF, Ni WF, Wang XY (2014). The relationship between vitamin A and risk of fracture: meta-analysis of prospective studies. J Bone Miner Res.

